# Can orbital angle morphology distinguish dogs from wolves?

**DOI:** 10.1007/s00435-015-0294-3

**Published:** 2015-12-30

**Authors:** Luc Janssens, Inge Spanoghe, Rebecca Miller, Stefan Van Dongen

**Affiliations:** Department of Archaeology, Leiden University, Einsteinweg 2, 2333 CC Leiden, The Netherlands; Hortensialaan 20, 2840 Reet, Belgium; Service of Prehistory, University of Liège, Quai Roosevelt, 1, 4000 Liège, Belgium; Department of Evolutionary Ecology, University of Antwerp, Groenenborgerlaan 171, 2020 Antwerp, Belgium

**Keywords:** Morphology, Dog, Wolf, Archaeology, Orbital angle

## Abstract

For more than a century, the orbital angle has been studied by many authors to distinguish dog skulls from their progenitor, the wolf. In early studies, the angle was reported to be different between dogs (49°–55°) and wolves (39°–46°). This clear difference was, however, questioned in a more recent Scandinavian study that shows some overlap. It is clear that in all studies several methodological issues were unexplored or unclear and that group sizes and the variety of breeds and wolf subspecies were small. Archaeological dog skulls had also not been studied. Our goal was to test larger and more varied groups and add archaeological samples as they are an evolutionary stage between wolves and modern dogs. We also tested the influence of measuring methods, intra- and inter-reliability, angle symmetry, the influence of variations in skull position and the possibility of measuring and comparing this angle on 3D CT scan images. Our results indicate that there is about 50 % overlap between the angle range in wolves and modern dogs. However, skulls with a very narrow orbital angle were only found in wolves and those with a very wide angle only in dogs. Archaeological dogs have a mean angle very close to the one of the wolves. Symmetry is highest in wolves and lowest in archaeological dogs. The measuring method is very reliable, for both inter- and intra-reliability (0.99–0.97), and most skull position changes have no statistical influence on the angle measured. Three-dimensional CT scan images can be used to measure OA, but the angles differ from direct measuring and cannot be used for comparison. Evolutionary changes in dog skulls responsible for the wider OA compared to wolf skulls are mainly the lateralisation of the zygomatic process of the frontal bone. Our conclusion is that the orbital angle can be used as an additional morphological measuring method to discern wolves from recent and archaeological dogs. Angles above 60° are certainly from recent dogs. Angles under 35° are certainly of wolves.

## Introduction

The domestication of wolves into dogs is currently actively debated (Boudadi-Maligne and Escarguel 2014; Germonpré et al. 2012; Larson and Burger 2013; Larson et al. 2012; Morey 2014). Where and when dogs originated and how to distinguish dog remains from those of wolves have been investigated by both osteo-archaeologists (morphologists) (Aaris-Sørensen 1977, 2004; Benecke 1987, 1994; Boudadi-Maligne and Escarguel 2014; Huxley 1880; Iljin 1941; Köhler and Moyà-Solà 2004; Nehring 1888; Rütimeyer 1861, 1875; Stockhaus 1965; Studer 1901; Sumiński 1975; Von Den Driesch 1976) and geneticists (Boyko et al. 2010; Druzhkova et al. 2013; Freedman et al. 2014; Gundry et al. 2007; Leonard et al. 2002; Natanaelsson et al. 2006; Savolainen et al. 2002; Vonholdt et al. 2010). In essence three morphological methods have been used: classical morphology and thus observable differences in form (Olsen and Olsen 1977), classical morphometry: measuring sizes and ratios (Benecke 1994; Morey 1986, 1992; Wayne 1986; Wolfgram 1894) and more recently, geometric morphometrics (Pionnier-Capitan 2010). These methods describe objective differences between the two sub-species and help to determine whether archaeological skulls belonged to wolf or dog. As such they contribute significantly to the question of when and where dogs were domesticated.

**Fig. 1:**
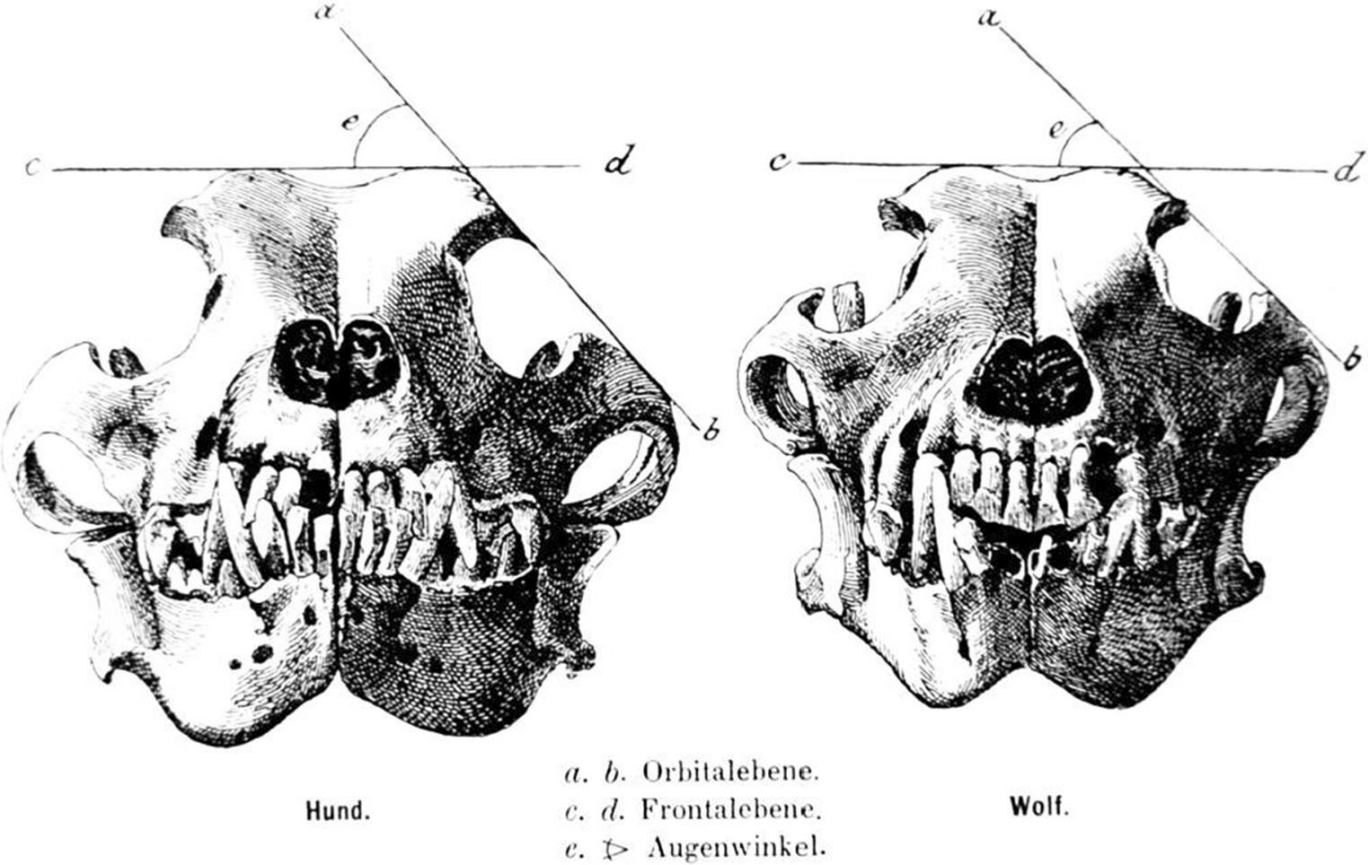
OA as depicted in the original Studer (1901) publication (Figure 1, p. 4). The skull is seen from rostral view. *Left* dog, *right* wolf skull. The OA is the dorsal angle between a horizontal leg on *top* of the frontal bones and tan oblique leg. The oblique leg can be drawn in two different ways: the dorsal contact point is identical in both (ZP), and the ventral contact point is the most lateral structure of two points to come in contact with “the measuring plane”. This ventral point can be the most dorsolateral point of the zygomatic arch (ZA) as in the dog skull, or the FP as shown in the wolf skull

**Fig. 2:**
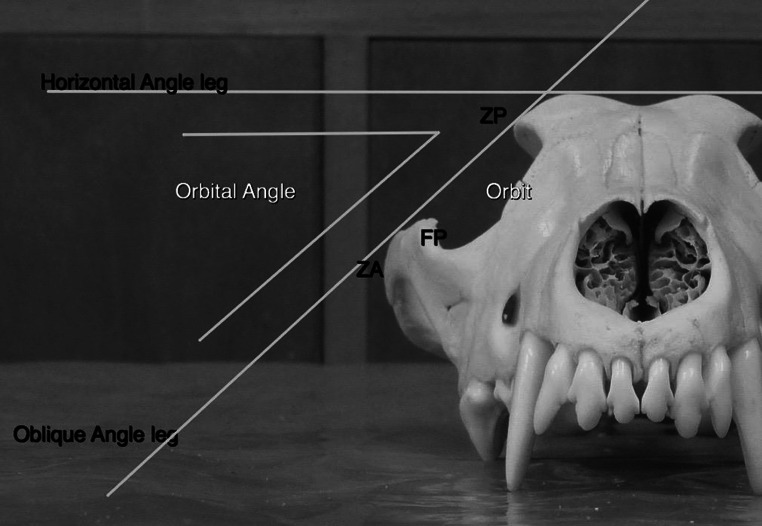
*Horizontal line* on *top* of the frontal bones represents the first leg of the angle. The oblique leg of the angle can be drawn in two ways: the dorsal contact point is stable (ZP) and the ventral contact point is the most lateral structure to contact “the measuring plane”, this is either ZA (as in this skull) or FP

**Fig. 3:**
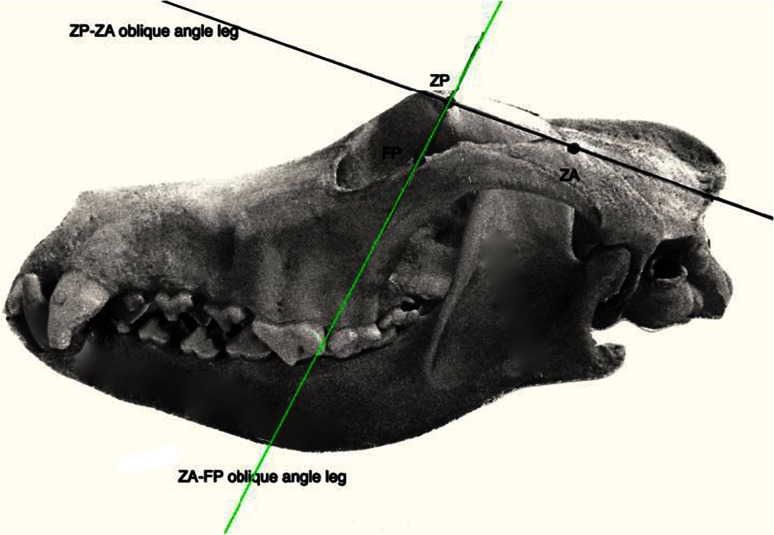
Lateral skull view. The almost *vertical line* represents the oblique leg of the OA when FP and ZP are contact points. The almost *horizontal line* represents the oblique leg of the OA when FP and ZA are contact points

The most important morphological and morphometric differences used to distinguish dogs from wolves and regarding dogs are: smaller stature, shorter and wider snouts, shorter carnassials, tooth crowding and wider orbital angles (Benecke 1987; Clutton-Brock 1962; Degerbøl 1961; Stockhaus 1965; Studer 1901; Wolfgram 1894). The orbital angle (OA) is a morphological ratio that depends on the width and height of specific skull landmarks. The method was developed by Studer and applied by observing the skull in rostral view (Fig. 1) (Studer 1901). Studer described the first leg of the angle as a horizontal line on top of the frontal bones. The second leg was defined by “placing a plane (*Ebene*) against the lateral side of the skull, in contact with two points of the orbita (*Augenrand*)”. Studer (1901) defined the upper contact point of the oblique leg as the most lateral point of the zygomatic process of the frontal bone (ZP), while the most ventral contact point was described as the most dorsolateral point of the frontal process of the zygomatic arch (FP) (Figs. 1, 2). The right figure in the original publication shows exactly the method as described in words (Fig. 1). The left figure, however, shows “another story” (Fig. 1). Here, the oblique leg is in contact with a different landmark at the ventral side: the dorsal rim of the zygomatic arch (ZA) (Figs. 1, 2) which is not part of the orbita and thus “sensu stricto” the use of this last point does not measure a “real” *Orbital* angle. While the difference between both ventral contact points may seem minimal seen in rostral view, there is a huge difference when seen from lateral (Fig. 3). Also, if ZA is used as the ventral contact point, the OA will be narrower compared to when FP would be used in the same skull. The reason for the two possible ventral contact points lies in the use of the “*Ebene*”: this plane touches the skull at the widest of the two anatomical structures. Studer does not report this difference.

Studer (1901) measured 21 wolf (19 Eurasian) and 24 dog skulls (>20 breeds) with 1° precision. Several other authors measured the OA with 0.5° precision; they were: Bockelmann (1920) who measured four Eurasian wolves and three German shepherds, Iljin (1941) who measured four Eurasian wolves and four German shepherds, Aaris-Sørensen (1977) who measured 35 Eurasian wolves (of which some were sub-fossils) and 35 dogs from three different breeds (including nine German shepherds) and Sablin and Khlopachev (2002) who measured two prehistoric putative dogs from Eliseevichi (Aaris-Sørensen 1977; Bockelmann 1920; Iljin 1941; Sablin and Khlopachev 2002). None of the authors after Studer (1901) described their methodology in detail. As most of these studies used Eurasian wolves only and mainly German shepherds as dogs, the variability of groups was very narrow. Most studies found distinct OA differences between wolves (39.5°–46.5°) and dogs (49°–55°). Aaris-Sørensen (1977) did, however, describe wider ranges in dogs (44°–56°) and wolves (36°–49°). He also reported an overlap (44°–49°), contrary to his predecessors, making the method less reliable and more restricted in use. Many methodological facets were clearly unreported in the former studies: Was an OA measured once or more often? Were means reported? Were all measurements performed by only one researcher and was intra- and inter-reliability considered? What was the difference in degrees when using the two different oblique legs of the angle? And in what percentage does one measure the “sensu stricto” OA? Was the angle measured on one or both sides of the skull and was fluctuating asymmetry taken into account? Perfect symmetry (defined as stability) is considered to be perfection of development. Failure to achieve symmetry is called developmental instability. Symmetry informs on the potential of the organism (both individuals and groups) to cope and channel its development during growth in an imperfect environment (Van Dongen 2006) and thus on the amount of environmental stress, and on the (partially hereditary) fitness of the organism. A higher degree of symmetry is observed in more dominant (higher in hierarchy) and more sexually attractive individuals with more offspring (De Coster et al. 2013). Species that originated recently show higher degrees of asymmetry than long existing species that have had time to develop higher developmental stability (De Coster et al. 2013; Van Dongen 2006; Van Dongen et al. 2009). For this aspect it might be of interest to compare symmetry in modern dogs (a modern species), wolves (a long existing species) and archaeological dogs (an early domesticated species).

Our aim in the present study was to address the methodological questions listed above and also to examine whether measuring larger groups with more variability (breeds and wolf subspecies) would change the published results. Another objective was to examine OA in archaeological dog skulls and compare these results to modern dogs and to their progenitor, the wolf. We were also interested in testing whether OA could be measured on 3D-CT scan images to provide results consistent with normal measuring results. If so, this would allow future scientists to examine digital images instead of actual skulls that are valuable and fragile. Ultimately, we hoped to explain what anatomical landmarks are responsible for a narrower OA in wolves and to determine the possible value of this morphological method for further use.

## Materials and methods

### Materials

#### Modern dog skulls

A total of 384 dog skulls, belonging to 71 breeds and 5 crossbreed dogs, were measured. These belong to the collection of the Department of Anatomy, Faculty of Veterinary Medicine Ghent, University Ghent, Belgium (123 skulls) and the collection of the Natural History Museum Bern, Switzerland (NMBE) (261 skulls) (Table 1).

**Table 1:** List of modern dog breeds used in this study

Breed	Nr	Breed	Nr
Afghan hound	13	Greyhound	10
Airdale terrier	4	Groenendael B. shepherd	18
Akita Inu	8	Hahoawu	1
Alaskan Malamute	5	Irish setter	2
Barzoi	11	Irish wolfhound	8
Basenji	1	Jagdterrier	2
Batak hound	11	Karelian Bear dog	32
Beagle	9	Kuvasc	1
Bearded collie	1	Labrador retriever	13
Berger de Brie	1	Leonberger	1
Berner sennenhund	32	Lundehund	2
Blood hound	7	Malinois Belgian shepherd	2
Border collie	5	Mastino Napolitano	1
Bouvier des Flandres	4	Mayar Agar	2
Boxer	2	Pariah hound	10
Bull terrier	1	Pembroke Welsh Corgi	1
Canaan dog	1	Pharaoh hound	4
Canadian Eskimo dog	4	Pointer	1
Chow Chow	16	Poodle	6
Cocker spaniel	4	Rhodesian Ridgeback	2
Crossbred	5	Rottweiler	3
Dalmatian	1	Saint Bernhard	2
Dingo	3	Saluki	2
Doberman pinscher	15	Samojeed	8
Entelbucher	1	Scottish collie	1
Finnish spitz	3	Scottish deerhound	2
Flatcoat retriever	1	Shar Pei	1
Fox terrier	1	Siberian Husky	14
Gaint schnauzer	1	Sloughi	1
Galgo Espanjol	2	Swiss shepherd	1
German braque	3	Tervueren Belgian shepherd	5
German shepherd	10	Tibetan Mastiff	6
Golden retriever	6	Tibetan terrier	1
Great Dane	2	Weimaraner	1
Great spitz	7	Whippet	4
Greenland dog	10	Wolf spitz	2
Total breeds *N*: 72; Total dogs: 384			

#### Archaeological dog skulls

Forty-five skulls were measured. Forty-three were from the ZMK_ZMUC collection of the Centre for GeoGenetics, Natural History Museum of Denmark, University of Copenhagen, Denmark. Most have not been dated by AMS C14, but rather by stratigraphy and associated artefacts. Most are attributed to the Boreal and Atlantic (Mesolithic) phase, while a few are of Neolithic age. Additionally, one Mesolithic skull was from the collection of LVR-Landes Museum, Bonn, Germany, and one from the Antikvarie collection, Osteology, Lund Universitets Historiska Museum, Lund, Sweden (Table 2).

**Table 2:** List of archaeological dog skulls used in this study

Country	NR	Location or ID	cal BP
Denmark	1	KATHALE BIRKEROD	
Denmark	2	?	
Denmark	3	?	
Denmark	4	TOVBROMEJERI RUBJERG	
Denmark	5	?	
Denmark	6	MARIUS PETERSENS MOLLEBY	
Denmark	7	?	
Denmark	8	OPKELSJET TROLDEBERG 1939	4600
Denmark	9	GRUNDOMAGEL DYBDE	
Denmark	10	GLUMSO VESTERGAARDS SORO	
Denmark	11	A 5446.2 HOVEDER	
Denmark	12	VKH 6215 X 63.62	
Denmark	13	STP	
Denmark	14	HERRINGLOSE	
Denmark	15	HYLLESTED	
Denmark	16	STEENSTRUP	
Denmark	17	HEDEHUSENE P 1040233	
Denmark	18	HAMMERSHOJ	
Denmark	19	HASMARK	3000
Denmark	20	HUNDSTRUD	
Denmark	21	KVAERKEBYBJERG	
Denmark	22	?	
Denmark	23	SKELLINGSTED	2000
Denmark	24	SNOLDELEV	5500
Denmark	25	TVEDGAARDM SKIBET	
Denmark	26	TIBIRKE	6500
Denmark	27	KAGMOSEN HUSUM	
Denmark	28	APPELDORN	
Denmark	29	BIRKEND	
Denmark	30	BRANDSTRUP	
Denmark	31	MAGLEMOLLE	
Denmark	32	MARREBAEK	
Denmark	33	MAGLEBRANDE	
Denmark	34	NONE	
Denmark	35	NYTORU	
Denmark	36	NYKOBING	
Denmark	37	NYBY	
Denmark	38	ORDRUP	
Denmark	39	RUBJERG	
Denmark	40	RISLEV	1600
Denmark	41	RANDERS	
Denmark	42	AALYKKESKOVEN	
Denmark	43	SENKENBURG-M4142-R 506_510	
Germany	44	BEDBURG	11,600
Sweden	45	SKATEHOLMGR/1674AVE 9	5400

Dating is C14 calibrated (calBP)

#### Recent wolf skulls

In total 55 skulls were measured. Thirty-eight (32 *Canis lupus pallipes* and 6 *C.l.arabs*) are from the collection of the George S. Wise Faculty of Life Sciences, Department of Zoology at Tel-Aviv University, Israel (ZMTAU) and seven (1 *Canis lupus pallipes* and 6 *C.l.arabs*) from the Natural History Museum, London, Great Britain (BMNH). Ten Eurasian skulls are from the Natural History Museum, Bern, Switzerland (NMBE) (Table 3).

**Table 3:** List of wolf skulls used in this study

Museum ID	Genus	Species	Subspecies	Region
BMNH ZD.1891.2.5.1	*Canis*	*lupus*	*arabs*	Bouraida
BMNH ZD.1895.10.8.1	*Canis*	*lupus*	*arabs*	Aden
BMNH ZD.1899.11.6.36	*Canis*	*lupus*	*arabs*	Muscat
BMNH ZD.1924.8.13.1	*Canis*	*lupus*	*arabs*	Jeddah
BMNH ZD.1940.193	*Canis*	*lupus*	*arabs*	?
BMNH ZD.1948.368	*Canis*	*lupus*	*pallipes*	?
BMNH ZD.1897.1.14.4	*Canis*	*lupus*	*arabs*	Jaquakar
NMBE1028185	*Canis*	*lupus*	*Lupus*	Russia
NMBE1028188	*Canis*	*lupus*	*Lupus*	Russia
NMBE1028189	*Canis*	*lupus*	*Lupus*	Russia
NMBE1028192	*Canis*	*lupus*	*Lupus*	Poland
NMBE1028193	*Canis*	*lupus*	*Lupus*	Russia
NMBE1028204	*Canis*	*lupus*	*Lupus*	Poland
NMBE1028205	*Canis*	*lupus*	*Lupus*	Poland
NMBE1028206	*Canis*	*lupus*	*Lupus*	Poland
NMBE1028207	*Canis*	*lupus*	*Lupus*	Poland
NMBE1028209	*Canis*	*lupus*	*Lupus*	Poland
ZMTAU 09439	*Canis*	*lupus*	*pallipes*	Golan
ZMTAU 09460	*Canis*	*lupus*	*arabs*	Sandiya
ZMTAU 10334	*Canis*	*lupus*	*pallipes*	Galilei
ZMTAU 10338	*Canis*	*lupus*	*pallipes*	Galilei
ZMTAU 10355	*Canis*	*lupus*	*pallipes*	Golan
ZMTAU 10402	*Canis*	*lupus*	*pallipes*	Golan
ZMTAU 10608	*Canis*	*lupus*	*pallipes*	Galilei
ZMTAU 10609	*Canis*	*lupus*	*pallipes*	Golan
ZMTAU 10610	*Canis*	*lupus*	*pallipes*	Goaln
ZMTAU 10615	*Canis*	*lupus*	*pallipes*	Golan
ZMTAU 10619	*Canis*	*lupus*	*pallipes*	Golan
ZMTAU 10621	*Canis*	*lupus*	*pallipes*	Golan
ZMTAU 10682	*Canis*	*lupus*	*pallipes*	Golan
ZMTAU 10685	*Canis*	*lupus*	*pallipes*	Golan
ZMTAU 10686	*Canis*	*lupus*	*pallipes*	Golan
ZMTAU 10688	*Canis*	*lupus*	*pallipes*	Golan
ZMTAU 10692	*Canis*	*lupus*	*pallipes*	Golan
ZMTAU 11041	*Canis*	*lupus*	*pallipes*	Galilei
ZMTAU 11109	*Canis*	*lupus*	*pallipes*	Galilei
ZMTAU 11110	*Canis*	*lupus*	*pallipes*	Golan
ZMTAU 11118	*Canis*	*lupus*	*pallipes*	Galilei
ZMTAU 11119	*Canis*	*lupus*	*pallipes*	Golan
ZMTAU 11121	*Canis*	*lupus*	*pallipes*	Golan
ZMTAU 11250	*Canis*	*lupus*	*pallipes*	Galilei
ZMTAU 11275	*Canis*	*lupus*	*pallipes*	Galilei
ZMTAU 11417	*Canis*	*lupus*	*pallipes*	Galilei
ZMTAU 11418	*Canis*	*lupus*	*pallipes*	Golan
ZMTAU 11475	*Canis*	*lupus*	*arabs*	Negev
ZMTAU 11476	*Canis*	*lupus*	*pallipes*	Golan
ZMTAU 11479	*Canis*	*lupus*	*pallipes*	Galilei
ZMTAU 11516	*Canis*	*lupus*	*pallipes*	Golan
ZMTAU 11685	*Canis*	*lupus*	*pallipes*	Golan
ZMTAU 12130	*Canis*	*lupus*	*pallipes*	Gallilei
ZMTAU 12130-2	*Canis*	*lupus*	*arabs*	Negev
ZMTAU 12251	*Canis*	*lupus*	*arabs*	Negev
ZMTAU 12254	*Canis*	*lupus*	*arabs*	Muscat
ZMTAU 12279	*Canis*	*lupus*	*arabs*	Negev
ZMTAU 11517	*Canis*	*lupus*	*pallipes*	Golan

BMNH, British Museum of Natural History; NMBE, Natural History Museum Bern and ZMTAU, George S. Wise Faculty of Life Sciences, Department of Zoology at Tel-Aviv University

### Methods

Mandible-free skulls were positioned in a horizontal plane with the midline aligned on the centre of the photographic lens. The skull was positioned as symmetrically as possible, but minor asymmetries <1° could not be excluded and not all skulls were anatomically perfectly symmetrical. Digital pictures were taken with a Nikon 700 digital camera with a stable objective off 50 mm focal lenght. The proximal tip of the lens was positioned at a distance of 40–50 cm from the most rostral point of the skull. Images were saved in RAW format and imported to the Dicom viewer of OsiriX MD software to measure OA. Software measurements were 0.001° but rounded off to a full degree. First, a horizontal line was drawn on top of the frontal bones as the first leg of the angle (Fig. 2). The oblique leg was drawn between ZP dorsally and ventrally the most lateral of two points: ZP or ZA (as described above) (Figs. 2, 3). Measurements were performed bilaterally and differences between sides were used in the symmetry study. Measuring intra- and inter-reliability was performed on 50 dog skulls each (100 measures) selected randomly and blindly, without knowledge of the results of the first measurements. Intra-reliability was performed by the second author (IS) and all other measurements by the first (LJ).

We opted in our study to use the plane technique and realised that this meant that in some cases ZA was used while in others FP was used as the ventral contact point of the oblique leg of the angle.

We considered that using the two different measuring methods could create a problem that would bias the results and hamper comparisons between groups. To address this issue, the percentage in which the oblique angle leg was in contact with ZP or ZA was calculated using 75 randomly selected skulls (25 from each group) of wolves, recent and archaeological dogs. The OA was measured in the standard way on both sides, and the measurement was then repeated using the ZP-FP contact points. The difference in OA was calculated between methods. The influence of imperfect skull position was measured in 5 dog skulls chosen randomly. OA was measured with a horizontal and symmetrical skull, with a 5° tilt upwards or downwards of the nose and with a 5° rotational deviation (right side forward). Asymmetry was measured in each individual skull and per group.

**Fig. 4:**
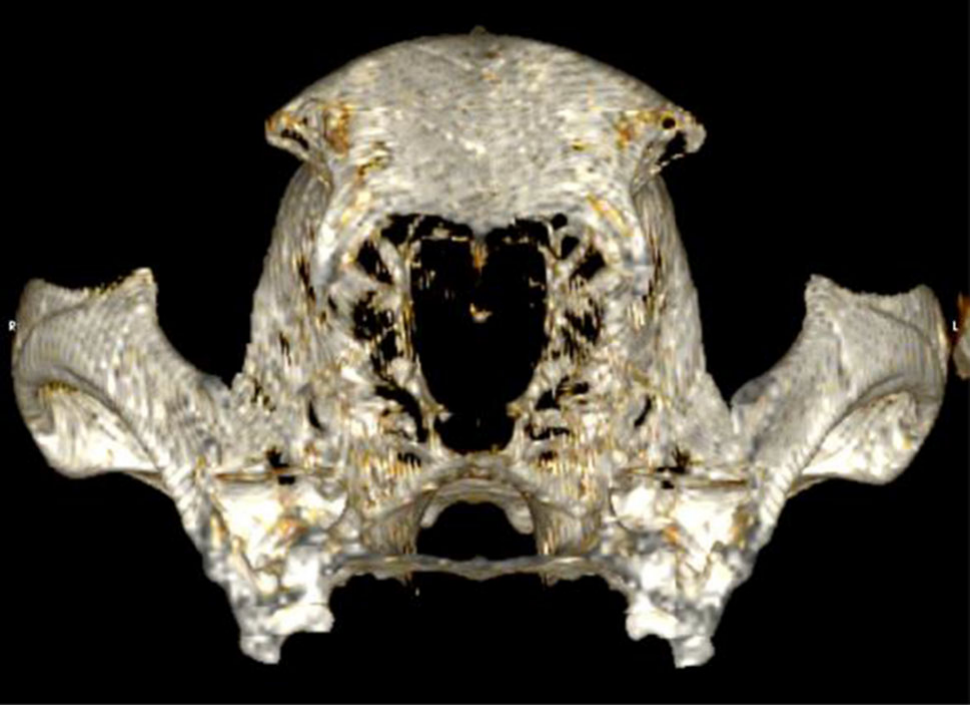
A 3D CT model reconstruction of a wolf skull in the OsiriX MD software program. Rostral view

Twelve skulls of the ZMTAU collection were scanned with a single slice Picker CT scan. Transversal 1-mm-thick slices were recorded. The DICOM images were imported into Osirix MD to create a 3D reconstruction (Fig. 4). The printout of this image was used to measure the OA with an orthopaedic goniometer.

ANOVA models, with individual as random effect, were used to test for statistical differences between groups, where the denominator degrees of freedom of the *F* test were approximated using the Satterthwaite method. Significance level was set at 0.01. To test reliability, individual was modelled as a random effect, which allowed us to measure inter-individual variation and intra-observer reliability.

## Results

**Fig. 5:**
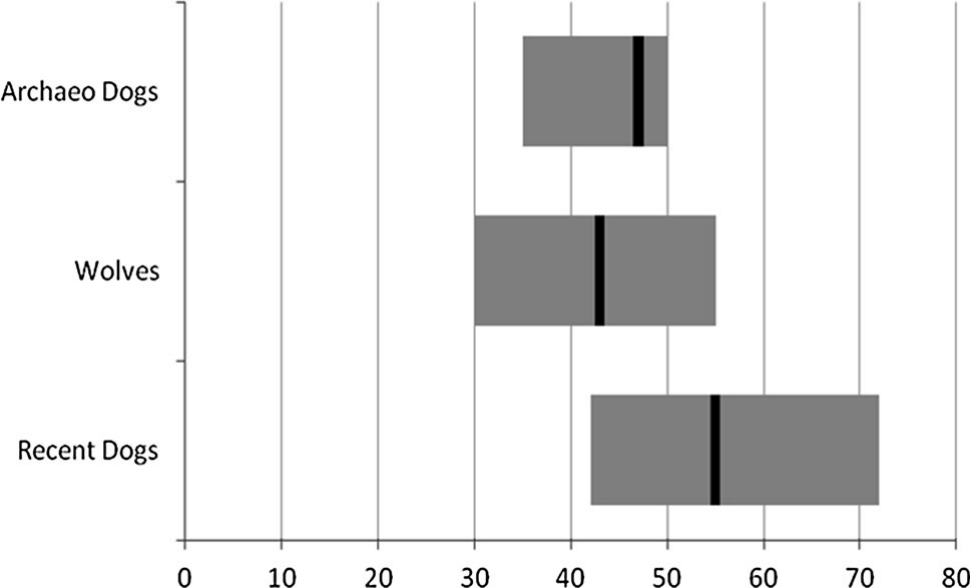
Block diagram of the mean OA and spread in recent and archaeological dogs and wolves

The mean OA in modern dogs was 55° (SD 5.8°, minimum 41°, maximum 72°). In archaeological dogs it was 47° (SD 4.7°, minimum 35°, maximum 60°) and 42° in wolves (SD 5.3°; minimum 28°, maximum 52°) (Fig. 5) (Table 4). These values were significantly different between recent and archaeological dogs (*p* < 0.0001), modern dogs and wolves (*p* < 0.0001), and wolves and archaeological dogs (*p* = 0.01).

**Table 4:** Overview of minima, maxima and means of the OA in modern and archaeological dogs and wolves

OA	Modern dogs	Wolves	Archaeological dogs
N	384	55	45
Min	41	28	35
Mean	55	42	47
Max	72	52	60
<35°	0 %	9 %	0 %
35°–52°	50 %	91 %	90 %
>52°	50 %	0 %	10 %

Also the overlap between categories is presented

The overlap between modern dogs and wolves ranged between 41° and 52°. In this range were 43 % of measurements of modern dogs and 61 % of wolves. The overlap zone of all dogs and wolves ranged between 35° and 60° and was present in 48 % of the dogs and 91 % of the wolves. The percentage of wolves that had an OA lower than 35° was 9 %. The percentage of recent dogs that had an OA larger than 52° was 56 % (Table 4).

Asymmetry in OA dog skulls was 1.7° (SD 1.8°) (*p* < 0.1), in wolves 1.2° (SD 1.3°) (*p* < 0.1) and in archaeological dog skulls 2.3° (SD 1.8°) (*p* < 0.1). Fluctuating asymmetry was significant between wolves and both dog groups (*F*_2,467_ = 5.07, *p* = 0.007) but not between archaeological and modern dogs (*p* = 0.17). Asymmetry was larger in archaeological dogs (mean 2.29°, SD 0.27) compared to modern dogs (mean 1.69°, SD 0.08, *p* = 0.04) and wolves (mean 1.23°, SD 0.20, *p* = 0.005). The inter-observer reliability was 0.99. The mean difference between the two researchers was between 0° and 2°. The intra-observer reliability was 0.97. The mean difference between the two measuring sessions was 1.2° with a range of 0°–7°.

The oblique leg of the angle was in contact with ZA as the ventral point in 92/100 wolves, 90/100 archaeological dog and 64/100 modern dog measurements. The difference between wolves and recent dogs was statistically significant (*χ*^2^ = 35.7, *d.f.* = 1, *p* < 0.0001), but no difference was observed between wolves and archaeological dogs (*χ*^2^ = 0.06, *d.f.* = 1, *p* = 0.80). The OA, measured in the subgroups with ZA as the ventral contact point, was 42° in wolves, 47° in archaeological dogs and 53° in modern dogs. Re-measuring the same skulls with FP as the ventral contact point resulted in an OA of 47° in wolves (gain 5°), 50° in archaeological dogs (gain 3°) and 59° in modern dogs (gain 4°). This gain in OA was statistically significant in the three groups (*F*_1,25_ = 220, *p* < 0.0001). The OA difference in the nose up position was 0.3° (*p* = 0.85) and nose down position 2.7° (*p* = 0.09). When rotating the skull position the OA decreased at the proximal side −1.1° (*p* = 0.56) and enlarged at the caudal side 7.3° (*p* = 0.0005).

The difference between measuring the same skulls with the standard method or in 3D CT scan images was 3° (0°–8°), a difference that was statistically significant (*F*_1,27_ = 26.0, *p* < 0.0001). Nevertheless, there was a positive correlation between individuals measured with both methods (*r* = 0.67).

Asymmetry in OA dog skulls was 1.7° (SD 1.8°) (*p* < 0.1), in wolves 1.2° (SD 1.3°) (*p* < 0.1) and in archaeological dog skulls 2.3° (SD 1.8°) (*p* < 0.1). Fluctuating asymmetry was significant between wolves and both dog groups (*F*_2,467_ = 5.07, *p* = 0.007) but not between archaeological and modern dogs (*p* = 0.17). Asymmetry was larger in archaeological dogs (mean 2.29°, SD 0.27) compared to modern dogs (mean 1.69°, SD 0.08, *p* = 0.04) and wolves (mean 1.23°, SD 0.20, *p* = 0.005). The inter-observer reliability was 0.99. The mean difference between the two researchers was between 0° and 2°. The intra-observer reliability was 0.97. The mean difference between the two measuring sessions was 1.2° with a range of 0°–7°.

The oblique leg of the angle was in contact with ZA as the ventral point in 92/100 wolves, 90/100 archaeological dog and 64/100 modern dog measurements. The difference between wolves and recent dogs was statistically significant (*χ*^2^ = 35.7, *d.f.* = 1, *p* < 0.0001), but no difference was observed between wolves and archaeological dogs (*χ*^2^ = 0.06, *d.f.* = 1, *p* = 0.80). The OA, measured in the subgroups with ZA as the ventral contact point, was 42° in wolves, 47° in archaeological dogs and 53° in modern dogs. Re-measuring the same skulls with FP as the ventral contact point resulted in an OA of 47° in wolves (gain 5°), 50° in archaeological dogs (gain 3°) and 59° in modern dogs (gain 4°). This gain in OA was statistically significant in the three groups (*F*_1,25_ = 220, *p* < 0.0001). The OA difference in the nose up position was 0.3° (*p* = 0.85) and nose down position 2.7° (*p* = 0.09). When rotating the skull position the OA decreased at the proximal side −1.1° (*p* = 0.56) and enlarged at the caudal side 7.3° (*p* = 0.0005). The difference between measuring the same skulls with the standard method or in 3D CT scan images was 3° (0°–8°), a difference that was statistically significant (*F*_1,27_ = 26.0, *p* < 0.0001). Nevertheless, there was a positive correlation between individuals measured with both methods (*r* = 0.67).

## Discussion

As the range in our study was 44°, measurements were rounded off to 1°, based on the statistical 30–300 rule (Heath 2002). Reliabilities were also very high with a 1°–2° difference in measuring, so there was no need to apply more precise measurement.

Our study demonstrates that archaeological dogs have OA values between those of dogs and wolves and are closer to wolves than modern dogs. In addition, the maximal OA in archaeological dogs lies closer to that of wolves: 50°–60°, not modern dogs (72°) (Table 4). Such OA values of archaeological dogs fit nicely into the evolutionary pathway, with wolves (mean OA 42°) as the progenitor to archaeological dogs (mean OA 47°) and the latter as the forefathers of modern breeds (mean OA 55°). However, things may be more complex. Indeed, most archaeological dog skulls were from a single geographical area (Scandinavia), and thus this group was probably geographically isolated for a long time and therefore closely related and morphologically similar. The OA of these archaeological dogs may thus not represent an evolutionary stage, but rather a genetically isolated population which had a coincidental OA close to wolves.

An interesting finding in this field comes from the study of Aaris-Sørensen (1977) who measured a statistically different OA in archaeological versus recent wolf skulls (resp. 44° and 41°) (*t* = 2.76, *d.f.* *=* 33, *p* = 0.01). This finding is contrary to the finding in dogs in our study, where more recent specimens have a higher OA. It might be that the difference in the study of Aaris-Sørensen (1977) is coincidental, but could, however, also be genetic or climate driven. If climate driven, it is difficult to explain why the OA in dogs and wolves would evolve following contrary paths. In our study, the OA dispersion is 28°–72° (44°) which is considerably more than the 16° scatter range published in earlier publications (Aaris-Sørensen 1977; Bockelmann 1920; Iljin 1941; Studer 1901). Further, the OA overlap between modern dogs and wolves is larger in our study than reported in the Aaris-Sørensen (1977) study (11° vs. 5°). This difference is probably caused by the larger group of skulls examined, as well as by the larger variety in wolf subspecies and dog breeds.

It is quite important to realise that if only recent wolf and dog skulls would have been studied, the overlap would be about half, and thus about 50 % of skulls could with certainty have been assigned to one of both groups: wolves to the group with OA between 28° and 42° and recent dogs to the group between 42° and 72°. Adding the archaeological dog group narrowed the percentage of wolf skulls that could be separated from archaeological and recent dog skulls to 9 % a small amount but still applicable.

It could be that the percentage to discern archaeological wolves from archaeological dogs by measuring OA is larger, given that archaeological wolves in the study of Aaris-Sørensen (1977) had a larger OA than recent wolves (44° vs. 41°). To be certain of this statement one needs, however, more archaeological wolf skulls to be studied and also more geographical areas. Such archaeological samples should ideally be obtained from archaeological sites from 18,000 to 16,000 calBP across Europe and the near East (thus the Magdalenian across its range in Europe as well as the Epipaleolithic and Natufian in Turkey and the near East) to complement the more recent specimens from Mesolithic and Neolithic contexts, which should be supplemented as well by specimens in different regions. It would be useful as well to have wolf specimens from non-archaeological contexts for this period to compare wolf with those specimens in direct contact with human populations.

Interestingly, the two putative archaeological dog skulls from Eliseevichi (Sablin and Khlopachev 2002) had an OA of 47°, which is in the non-diagnostic overlap zone, but the archaeological Predmosti 1, OK 1062 specimen (Germonpré et al. 2012) which could not be classified in the original study had an OA of 32° (Germonpré et al. 2012, Figure 6, p. 191), thus very close to the lowest OA of wolves. Here this additional measure (the OA) could convincingly place this last specimen in the wolf group.

The range of OA in modern dog breeds is the largest of the three groups (42°–72°). It is tempting to believe that the great anatomical variability of modern dog breeds is the cause of this OA range and these very high angles. However, the highest OA measured in our study was in a Border collie. This is at least puzzling as this is a mesaticephalic, small shepherd breed with a very “normal” morphology on view, much like a small German shepherd, the breed so favoured by previous authors examining the OA, as it was assumed to be a primitive type breed, still very like wolves.

The highest amount of asymmetry was seen in archaeological dogs followed by modern dogs. Wolves were the most symmetrical. These findings fit nicely in the evolutionary development of the three groups with wolves being a long existing species and dogs having split from wolves about 18,000 years ago (Thalmann et al. 2013). Wolves should thus logically have the most symmetrical OA and archaeological dogs the least as this was “the new species” close in time to the source of its origin. As our archaeological dog skulls were estimated to be on average 6–7000 years old, they had split from wolves for about 11–12,000 years, while the modern dogs we examined had genetically split off from wolves 18,000 years ago, and thus had over a longer period of time to develop into a new species, and thus develop more symmetry. An argument to support this view is that the oldest specimen in our series, the *Bedburg* dog, had a very high asymmetry (4°), more than the mean of archaeological dogs (mean 2.29°, SD 0.27) (Table 2).

The discovery that Studer (1901) presented two different methods to measure the OA was unexpected. Re-measuring the skulls with the FP contact point resulted in a higher OA in the three groups (wolves +5°, archaeological dogs +3°, recent dogs +4°), and thus there was hardly any difference in discriminating the group means. This difference is not statistical (*F*_3,46_ = 0.90, *p* = 0.45), and thus both groups can be separated as well with the corrected method.

Positional differences had little influence on OA outcome. Only rotational asymmetry created a statistically different angle and only at the caudally translated side. These rotations were, however, extreme and incomparable with minimal asymmetries of some skulls examined in our series.

Why the OA widens can be explained mathematically by a lateral and/or downward shift of ZA (or FP) or an upward and/or inward shift of ZP (Fig. 2). One of these shifts or combinations could be possible. Studer (1901) carefully studied the question of wider OA in dogs and found that in equally large skulls of dog and wolf, with a 12° difference in OA, the total width of skulls measured at the widest point of the zygomatic arch (zygon–zygon) (measure 30) was identical (Von Den Driesch 1976). Therefore the lateral shift of ZA (or FP) could be excluded as the reason of a change in OA. Studer (1901) then realised that dog skulls had a wider ectorbitale–ectorbitale width (measure 32) (Von Den Driesch 1976) and thus a lateralisation of FP. The same conclusion, regarding the difference between wolves and modern dogs, came from the study of Aaris-Sørensen (1977). Moreover, he measured a 10 % increase in measure 32 in archaeological wolves when compared to recent wolves. That explained the larger OA he measured in these archaeological wolves (as described above).

Three recent studies report on anatomical changes between skulls of wolves and dogs. One study describes the enlarged orbital region (and thus FP lateralisation) in dogs when compared to wolves (Schmitt and Wallace 2012). A geometric morphometrics study in mesaticephalic dogs describes maxillar skull widening at the M1 level, which is at the rostral region of the orbital region (and thus FP lateralisation). Finally, a new study compared skulls of recent and archaeological dogs and wolves (Drake et al. 2015). In dogs, changes were shortening and angulation of the snout, rostral and upward movement of frontals (stop formation) and widening of the orbital region (and thus FP lateralisation). These recent studies confirm Studer’s (1901) conclusions from more than 100 years ago: the frontal process (FP) shifts to lateral and thus the OA widens.

To conclude, this paper examined five main aspects for the use of OA as a morphological method to distinguish between wolves and dogs: (1) methodological issues, (2) increased sample size, (3) use of 3D CT scan images, (4) identification of the anatomical landmarks responsible for a narrower OA in wolves and (5) evaluation of the potential value of this method. First, the use of FP instead of ZA as the ventral contact point results in statistically significant differences, with a gain in degrees in all three groups, the method is very reliable with respect to intra- and inter-reliability, symmetry is highest in wolves and lowest in dogs and skull position changes have no significant effect on the angle measured. Second, increased sample size showed a broader OA dispersion and a greater overlap between modern dogs and wolves than reported in other studies. Third, OA can be measured on 3D CT scans but cannot be compared with direct measuring results on actual skulls. Fourth, the wider OA in dogs is due to lateralisation of the zygomatic process of the frontal bone. Fifth, our conclusion is that the OA can be used as an additional morphological measuring method. Although its value to separate dog and wolf skulls is restricted to extreme values, a considerable amount of skulls fall into these categories.

In the future a wider geographic range of archaeological (pre- and post-LGM) and recent dogs ought to be measured, to confirm that archaeological dogs elsewhere are intermediate between wolves (ancient and recent) and modern dog breeds. This might be one aspect that helps to resolve recent claims that Gravettian canids are semi-domesticated “wolf-dogs”.
